# Micronutrient Supplementation and Deworming in Children with Geohelminth Infections

**DOI:** 10.1371/journal.pntd.0002920

**Published:** 2014-08-07

**Authors:** Selvi Rajagopal, Peter J. Hotez, Donald A. P. Bundy

**Affiliations:** 1 Departments of Medicine, Pediatrics, and Molecular Virology and Microbiology, National School of Tropical Medicine, Baylor College of Medicine, Houston, Texas, United States of America; 2 Sabin Vaccine Institute and Texas Children's Hospital Center for Vaccine Development, Houston, Texas, United States of America; 3 Human Development Department, World Bank, Washington, D.C., United States of America; University of Washington, United States of America

## Introduction

Soil-transmitted helminth (also known as “geohelminth”) infections are among the most common chronic infections worldwide. The World Health Organization (WHO) estimates that almost 900 million children require treatment (also known as deworming) for geohelminth infection, while the 2010 Global Burden of Disease Study estimates that more than 5.2 million disability-adjusted life years (DALYs) are attributable to geohelminth infection [1], [2]. In 2001, the World Health Assembly resolved to treat 75% of children at risk for morbidity from these geohelminths by 2010. However, WHO reported that by 2010 only approximately one-third of all children at risk had achieved access to mass drug administration (MDA). Treating the remaining two-thirds by 2020 is the target of the 2012 London Declaration for Neglected Tropical Diseases [3].

The 2012 London Declaration and the global aspirations for universal deworming arise partly from studies conducted over the last two decades demonstrating the severe morbidity and poor cognitive, intellectual, and physical child development in populations with endemic geohelminth infections, and the resulting economic losses [4], [5]. Nutritional deficiencies, including protein malnutrition and micronutrient losses secondary to geohelminth infection, represent a potentially important mechanism by which geohelminths weaken their host, causing ill health and disability [5]. There is growing evidence that serum levels of multiple micronutrients, including vitamin A, iron, copper, selenium, cobalt, and zinc, are reduced by geohelminth infection and some evidence that these effects can be reversed by deworming [6], [7]. In some cases, micronutrient supplementation has been added in order to complement local or regional deworming efforts.

Here we focus on iron and vitamin A deficiencies, two major micronutrient deficits which have been causally linked to geohelminthiases. We specifically explore the relative benefits of vitamin A supplementation for children with ascariasis infection and iron supplementation for children with trichuriasis and hookworm infections, with and without deworming, and consider whether there are circumstances in which deworming programs should be complemented by micronutrient programs.

## Vitamin A

Vitamin A deficiency is defined by WHO as a serum retinol level <0.35 mmol/L [8]. Several studies have linked human *Ascaris lumbricoides* infection to vitamin A deficiency with robust evidence for a relationship between high intensity ascaris infection and lower levels of vitamin A (<0.70 µmol/L) [9]. The relationships between trichuris and hookworm infections and vitamin A deficiency are less well documented; therefore, this discussion is limited to ascariasis.

Not all children with ascariasis meet WHO criteria for vitamin A deficiency, although serious health consequences have been observed in the children who still have relatively lower vitamin A levels (<0.70 µmol/L). Xeropthalmia, a severe complication of vitamin A deficiency that sometimes leads to blindness, is found more commonly in children with ascariasis [10]. As well as having direct effects, severe vitamin A deficiency can have significant indirect consequences, for example, increasing susceptibility to potentially fatal illnesses such as measles and lower respiratory infections [11]. As a result, the link between vitamin A deficiency and ascariasis has potentially important consequences for global health, especially since ascariasis may be the most common chronic childhood infection worldwide [12].

While the exact mechanism of deficiency induced by ascariasis remains unclear, studies have established that children absorb less vitamin A following oral supplementation when they are infected [13]. Mucosal changes in the gastrointestinal tract with ascariasis, including blunting of the intestinal villi and morphological changes in the intestinal crypts, may explain the malabsorption [14]. Similarly, the ability of the intestinal tract to absorb vitamin A, a fat-soluble vitamin, may be influenced by the impaired capacity for intestinal fat absorption in children with ascariasis infection [13].

Methods of improving vitamin A status in children have been explored in relation to supplementation and deworming, alone and in combination. There is extensive literature on the benefits of vitamin A supplementation alone on the community health of preschool-age pediatric populations, although a recent large-scale trial in India and associated meta-analysis of all large-scale trials suggests that the effect in terms of mortality prevention may be less than sometimes suggested [15]. In communities in which vitamin A deficiency coexists with geohelminth infection, deworming alone and in combination with vitamin A supplementation has been explored as a means to correct or reduce deficiency in infected children. A population study in Uttar Pradesh, India, found that twice-yearly deworming alone on lightly infected children did not result in either significant weight gain or a reduction in mortality [16]. Studies of preschool-age children in Bangladesh, India, and Indonesia, however, showed that combined deworming and supplementation with beta-carotene, an inactive form of vitamin A, resulted in the greatest rise in serum retinol levels, as compared to children treated with deworming alone or beta-carotene supplementation exclusively [17], [18]. While there is some evidence to suggest an added benefit to combined deworming and vitamin A supplementation, a number of studies in similar settings have either been unable to reproduce this positive outcome or have not specifically tested the combined intervention [19], [20]. Thus, while the WHO and the United Nations Children's Fund (UNICEF) recommend that benzimidazole anthelminthics can be safely coadministered with vitamin A [21], the potential synergies of deworming with vitamin A supplementation are neither well documented nor consistent. There is a need for additional studies, especially randomized clinical trials, given the frequent coexistence of dietary deficiency and infection in endemic areas.

## Iron

In contrast to its relationship with vitamin A, a strong association has not been identified between ascariasis infection and anemia. Most of the pediatric studies of geohelminth infection and the level of anemia have examined the relationship with either whipworms (*Trichuris trichiura*) or hookworms (*Necator americanus* or *Ancylostoma duodenale*). Most of these studies have examined subjects aged 2–15 years, in which anemia is defined as a hemoglobin concentration of <11.5 g/dL [22]. Moderate-to-heavy hookworm infection in children (and light hookworm infection in both pregnant and nonpregnant adults) and heavy pediatric trichuriasis can result in anemia, although the precise burden of infection that serves as a threshold for clinical anemia varies depending on the host's existing iron stores.

In the case of hookworm infection, iron deficiency anemia occurs when adult hookworms attach to the mucosa and submucosa, where they cause intestinal blood loss [23]. In a systematic review, Smith and Brooker showed that moderate-to-heavy-intensity hookworm infections were typically associated with low hemoglobin levels in school-age children [24]. Among adults, even light infections can produce anemia, especially in pregnant women [25]. In areas where hookworm transmission is intense, such as in Zanzibar, 25% of all anemia, 35% of iron-deficiency anemia, and 73% of severe anemia were attributable to hookworm [26]. In sub-Saharan Africa, hookworm and malaria coinfections are common and are often additive in terms of producing severe anemia [27]. Heavy infections with whipworm are also linked to anemia through a combination of blood loss as a result of trichuris dysentery syndrome and chronic inflammation (anemia of chronic disease) due to trichuris colitis [28], [29].

There is substantial evidence that iron-deficiency anemia from causes in early childhood can result in intellectual, cognitive, and behavioral deficits; several different mechanisms have been proposed, including altered dopaminergic function [30]. Moderate-to-heavy hookworm infections and trichuriasis specifically have in some circumstances been shown to lead to failure to achieve intellectual potential [31] and cognitive impairment [32]. Malaria occurring in combination with hookworm infection has been identified as a potential risk factor, exacerbating the cognitive deficits [33]. Anemia and a moderate-to-heavy parasite burden of either helminth species were identified as independent risk factors for stunting [34]. Both stunting and cognitive delay have been shown to have lifelong consequences for the productive potential of children.

In their systematic review of interventions to reduce hookworm anemia following deworming, Brooker and Smith determined that, for children and adults, albendazole had demonstrated benefit (a mean increase of 1.89 g/l), whereas the benefits from mebendazole did not reach statistical significance [35], findings that appear consistent with previous observations of higher hookworm cure rates from albendazole versus mebendazole [36]. Whether including iron supplementation with deworming adds substantially to the overall benefit is unclear. Brooker and Smith's systematic review found no added benefit of combining deworming with iron supplementation [35]. However, a study from Sierra Leone demonstrated additive benefit of deworming and iron-folate supplementation in pregnant women with hookworm [37], while both in Sri Lanka and India there was added benefit from adding mebendazole to iron supplementation [38], [39]. This seems to suggest that the effectiveness of MDA might be increased by routine iron supplementation of pregnant women, but further studies are required to determine whether iron supplementation is relevant to other populations. Such studies should also take into account the greater blood loss from *A. duodenale* compared to *N. americanus* [40] so that the benefits of iron supplementation in a given location may reflect the relative prevalence of these endemic hookworm species.

## Conclusions

More than a thousand million deworming treatments have been delivered for the three common (and coendemic) geohelminths. The evidence presented here suggests that adding micronutrient supplementation may provide additional benefits, but the case is far from clear. With vitamin A, there are demonstrable additive effects in some settings, but in others there is no clear benefit or no clear difference from supplementation alone. With iron supplementation, in contrast, there does not appear to be a significant added benefit over deworming alone except in two cases: first, in pregnant women in hookworm endemic areas and, second, in those with very intense infection and existing anemia. Individuals in both situations could benefit from a combination of deworming and supplementation.

Delivering a bundled package for deworming and micronutrient supplementation would have financial implications. These implications are potentially much less for vitamin A supplements than for iron since the delivery regime for the former is similar in frequency to deworming—once or twice a year. Thus, in theory they could be delivered together using the same delivery platform. However, current evidence does not provide clear guidance that this would be beneficial under most circumstances.

It would not appear practical to integrate deworming with iron supplementation since the latter requires an extended regime of daily or weekly supplementation over several weeks. Such an approach is currently adopted as part of clinical case management and is appropriate to the setting of antenatal care or the management of severe anemia. Thus, in populations in which hookworm is a significant hazard and MDA for deworming is offered, it would be useful to ensure that those responsible for case management in the community were aware of the particular synergies with iron supplementation, especially for pregnant women.

It is perhaps worth emphasizing that our hesitancy in recommending cointervention is because of practical and cost-efficiency issues and not because of the lack of evidence of a causal link between worm infection and micronutrient deficiency. Even if helminth infection is merely associated with risk of micronutrient deficiency, with no proven causation, then linking micronutrient supplementation to deworming programs would still be justified on the grounds that poor children would likely benefit from both interventions. Further operational studies are needed to explore how best to take advantage of this huge opportunity to target children at high risk for both conditions.

Adding supplementation to deworming programs might offer benefits, but the current state of evidence appears insufficient to justify the potentially significant additional costs and complexity. A firmer recommendation would require further randomized clinical trials across different epidemiologic settings.
